# Not Hot, but Sharp: Dissociation of Pinprick and Heat Perception in Snake Eye Appearance Myelopathy

**DOI:** 10.3389/fneur.2018.01144

**Published:** 2018-12-21

**Authors:** Jan Rosner, Michèle Hubli, Pascal Hostettler, Catherine R. Jutzeler, John L. K. Kramer, Armin Curt

**Affiliations:** ^1^Spinal Cord Injury Center, Balgrist University Hospital, University of Zurich, Zurich, Switzerland; ^2^International Collaboration on Repair Discoveries (ICORD), University of British Columbia, Vancouver, BC, Canada

**Keywords:** cervical myelopathy, snake eye myelopathy, contact heat evoked potentials (CHEPs), pinprick evoked potentials (PEPs), spinothalamic tract, clinical neurophysiology

## Abstract

Following a traumatic spinal cord injury, a 53-year-old male developed a central cord syndrome with at-level neuropathic pain. Magnetic resonance imaging revealed a classical “snake eye” appearance myelopathy, with marked hyperintensities at C5-C7. Clinical examination revealed intact pinprick sensation coupled with lost or diminished thermal/heat sensation. This dissociation could be objectively confirmed through multi-modal neurophysiological assessments. Specifically, contact heat evoked potentials were lost at-level, while pinprick evoked potentials were preserved. This pattern corresponds with that seen after surgical commissural myelotomy. To our knowledge, this is the first time such a dissociation has been objectively documented, highlighting the diagnostic potential of multi-modal neurophysiological assessments. In future studies, a comprehensive assessment of different nociceptive modalities may help elucidate the pathophysiology of neuropathic pain.

## Introduction

The spinothalamic system transmits noxious and innocuous thermal and noxious mechanical information. While the assessment of thermo-nociceptive pathways using contact heat evoked potentials (CHEPs) is well-established for the diagnosis of incomplete spinal cord lesions (1, 2), objective readouts for pinprick evoked pain (pinprick evoked potentials [PEPs]) are novel (3).

Examination of the different modalities of nociception is recommended for a comprehensive assessment of spinothalamic integrity (3). Moreover, residual sparing of specific modalities in discomplete spinal cord lesions has been related to the development of central neuropathic pain (4). In the present case study, multi-modal electrophysiological testing, including CHEPs and PEPs, were performed in a patient with a cervical “snake eye” appearance myelopathy.

The specific aim was to compare segmental outcomes, neuroimaging, and clinical signs and symptoms.

## Case Presentation

A 53-year-old male with a posttraumatic incomplete spinal cord injury (AIS D, sub C4) was examined during his neurological rehabilitation at the Spinal Cord Injury Center at Balgrist University Hospital, Switzerland. The study was approved by the “Kantonale Ethikkommission Zürich” (EK-04/2006 / PB_2016-02051, clinicaltrial.gov number: NCT02138344) and written informed consent for publication was obtained from the patient.

**Figure 1:**
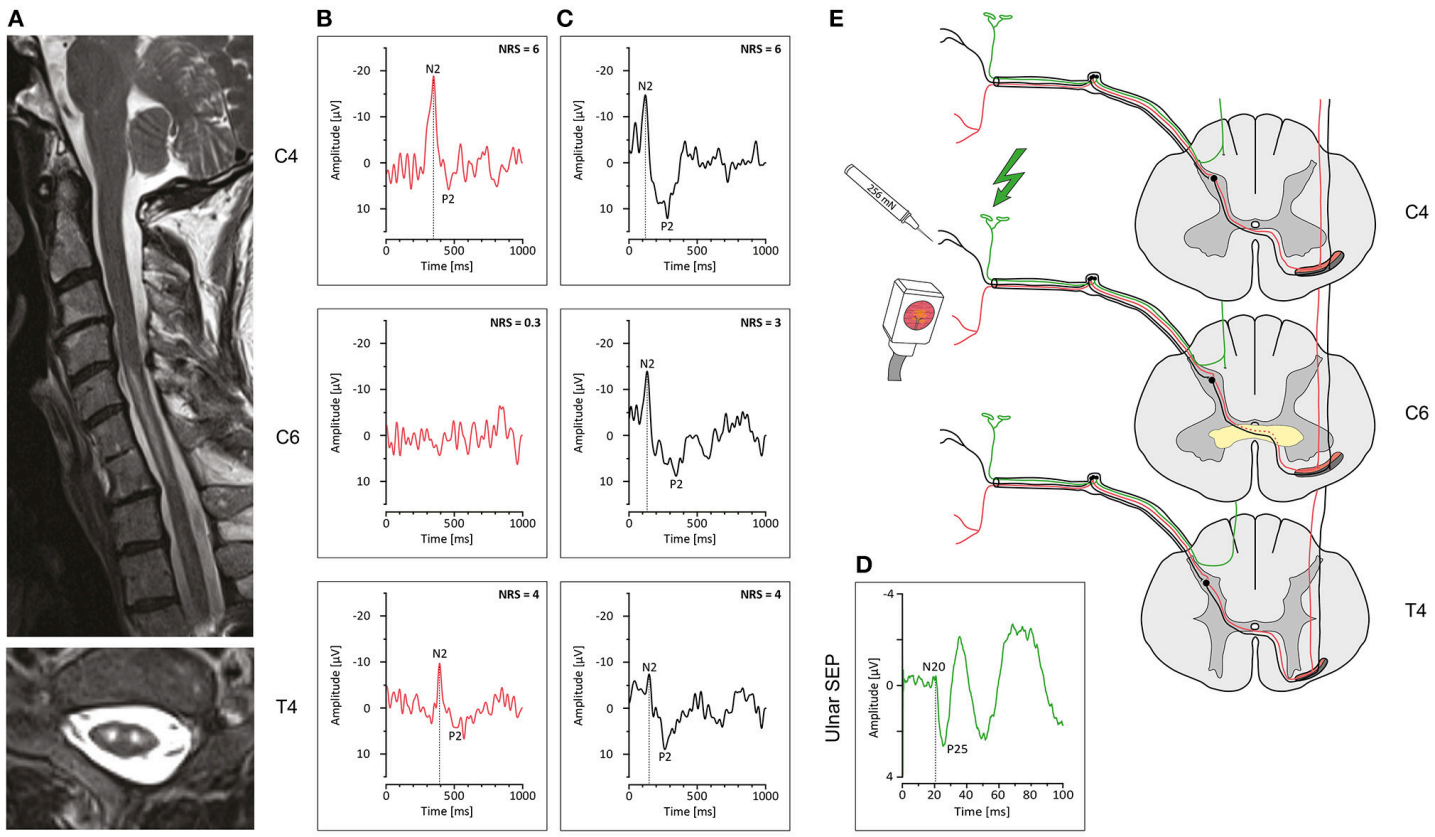
Neuroimaging and electrophysiological findings in “snake eye” appearance myelopathy. **(A)** Sagittal T2w image (above) and axial T2w image at C6 depicting a signal hyperintensity (“snake eye” appearance) located bilaterally in the central cord spanning about 3 segments. **(B)** Contact heat evoked potentials recorded at C4, C6, and T4 on the left, with an absent response at C6. **(C)** Pinprick evoked potentials recorded at C4, C6, and T4 on the left, with responses from all segments. **(D)** Somatosensory evoked potential recorded from the left ulnar nerve. **(E)** Schematic drawing indicating a spinal lesion (yellow) located around the spinal anterior commissure impairing conduction of crossing heat-sensitive pathways (dotted red line) while sparing noxious mechanosensitive pathways (black line). The color coding of the antero-lateral spinothalamic tract positions mechanical afferents (black) lateral and superficial to thermal afferents (red). Dorsal-column pathways are unaffected by the anteriorly located lesion (green line). Normal conduction is shown for supra- and infra-lesional stimulation indicating preservation of the antero-lateral quadrant where the spinothalamic tract ascends.

The patient presented clinically with a central cord syndrome, and at-level neuropathic pain with mechanical allodynia (i.e., brush allodynia) on both forearms. Light touch was normal within the cervical segments, and sharp dull discrimination was preserved while the intensity of the pinprick stimulus was only mildly attenuated. The upper extremity motor score (left: 11/25, right: 11/25) showed a profound weakness of elbow extensors, and no voluntary muscle activity in the finger flexors and abductors bilaterally. The lower extremity motor score was normal (left: 25/25, right: 25/25). Biceps and brachioradialis reflexes, as well as knee-jerk and ankle-jerk reflexes were bilaterally exaggerated. The Babinski sign was negative. Muscle tone was normal without signs of spasticity. Coordination and gait showed discrete signs of ataxia. Magnetic resonance images revealed a multi-segmental (C5-C7), longitudinal “snake eye” appearance myelopathy (Figure 1A). Ulnar somatosensory evoked potentials (SEPs) and sensory nerve conduction studies were normal (Figure 1D).

The CHEP recording was performed according to an established protocol (1, 2). Noxious mechanical stimuli were applied with a 256 mN pinprick stimulator (MRC Systems, Heidelberg, Germany). Stimulus application was performed in a slow fashion in order to favor recruitment of A-delta fibers (5). The skin was stimulated 20 times from a distance of 1 cm within 1 s. The experimenter was trained prior to the experiment to guarantee the stimuli are uniformly applied.

CHEPs were normal at C4 and T4 with high pain ratings to the heat stimulation, but almost complete loss of painful heat sensation and absent CHEPs at the level of C6 (Figure 1B). In contrast, PEPs remained preserved at all segments (Figure 1C).

## Discussion

The patient presented with signs and symptoms of a posttraumatic central cord syndrome including at-level mechanical allodynia. MRI findings indicated a “snake eye” appearance myelopathy with bilateral high intensity signal changes in the central gray matter from below C5 down to C7. Corresponding with radiographic findings, the patient showed a clinical dissociation of normal light touch (corresponding to normal ulnar SEPs) but mildly impaired pinprick sensation. However, cervical CHEPs revealed a distinct impairment of noxious heat sensation, while mechanically evoked potentials (i.e., PEPs) remained normal throughout all tested segments. Below the level of the focal central myelopathy heat sensation and CHEPs remained normal.

Collectively, these observations confirm pathology in decussating spinothalamic afferents conveying heat sensation, coupled with preserved integrity in the antero-lateral quadrant and dorsal columns (Figure 1E). Unlike CHEPs, PEPs were preserved in all spinal segments (i.e., above, at, and below level) and the findings reveal discrete centro-medullary pathology involving the anterior commissure related to “snake eye” myelopathy.

A previously published case determined that PEPs are conducted in the antero-lateral system, abolished alongside laser evoked potentials in a patient with a selective lesion of the contralateral anterolateral quadrant (3).

Corresponding sensory deficits associated with antero-lateral cordotomy include the loss of both pinprick and temperature sensations (6). Midline damage of the spinal cord, interrupting conduction in decussating spinothalamic afferents bound for the contralateral antero-lateral tracts (i.e., through commissural myelotomy), yields similar sensory deficits, including the loss of temperature sensation. Interestingly, however, pinprick sensation often remains intact (7).

The patient reported herein presented with sensory deficits consistent with midline spinal cord damage (i.e., loss of temperature sensation and preserved pinprick and light touch sensation). These findings were confirmed by neuroimaging and through the application of multi-modal electrophysiological testing—the latter demonstrating objective signs of preserved pinprick and absent temperature sensation at C6. Based on damage localized to the centro-medullary cord in this patient, one potential explanation for the dissociation between temperature and pinprick sensation is a distinct topographical organization within the decussating spinothalamic pathways. Afferents conveying temperature sensation are positioned posterior to those afferents conveying pinprick sensation (Figure 1E). This would, in principal, correspond with the representation of mechanical and temperature pain in the ascending antero-lateral tracts (6), and allow pinprick afferents to bypass the centro-medullary spinal cord pathology.

The present case highlights the application of multi-modal neurophysiological assessments combined with conventional neuroimaging to improve the characterization of spinal cord damage beyond conventional clinical examination. Their combined application may also play an important role in elucidating novel mechanisms underlying the development of neuropathic pain.

## Author Contributions

JR contributed substantially to the conception and design of the study, the data acquisition, analysis, and interpretation. Furthermore, he was chiefly involved in drafting the manuscript. MH contributed substantially to the data acquisition and interpretation, and revised the research article. PH was chiefly involved in creating the figure and contributed to data acquisition and analysis. CJ was involved in the data analysis and revising the research article. JK was involved in the data analysis and contributed substantially to the revision of the research article for important intellectual content. AC contributed substantially to the conception and design of the study, data analysis and interpretation, and was involved in revising the research article for important intellectual content.

### Conflict of Interest Statement

The authors declare that the research was conducted in the absence of any commercial or financial relationships that could be construed as a potential conflict of interest.

## References

[1] Ulrich A Haefeli J Blum J Min K Curt A. Improved diagnosis of spinal cord disorders with contact heat evoked potentials. Neurology (2013) 80:1393–9. 10.1212/WNL.0b013e31828c2ed1 23486867

[2] Jutzeler CR Ulrich A Huber B Rosner J Kramer JL Curt A. Improved diagnosis of cervical spondylotic myelopathy with contact heat evoked potentials. J Neurotrauma (2017) 34:2045–53. 10.1089/neu.2016.4891 28260398

[3] Iannetti GD Baumgartner U Tracey I Treede RD Magerl W. Pinprick-evoked brain potentials: a novel tool to assess central sensitization of nociceptive pathways in humans. J Neurophysiol. (2013) 110:1107–16. 10.1152/jn.00774.2012 23678019

[4] Wasner G Lee BB Engel S McLachlan E Residual spinothalamic tract pathways predict development of central pain after spinal cord injury. Brain (2008) 131(Pt 9):2387–400. 10.1093/brain/awn169 18669485

[5] van den Broeke EN Mouraux A Groneberg AH Pfau DB Treede RD Klein T. Characterizing pinprick-evoked brain potentials before and after experimentally induced secondary hyperalgesia. J Neurophysiol. (2015) 114:2672–81. 10.1152/jn.00444.2015 26334010 PMC4644227

[6] Taren JA Davis R Crosby EC. Target physiologic corroboration in stereotaxic cervical cordotomy. J Neurosurg. (1969) 30:569–84. 10.3171/jns.1969.30.5.0569 4889537

[7] Hitchcock E. Stereotactic cervical myelotomy. J Neurol Neurosurg Psychiatry (1970) 33:224–30. 4910200 10.1136/jnnp.33.2.224 PMC493447

